# Pediatric pleural empyema - decision making beyond imaging - a retrospective cohort study

**DOI:** 10.3389/fped.2026.1781021

**Published:** 2026-05-29

**Authors:** Alexandru-Ioan Ulmeanu, Andreia Ulmeanu, Elena-Roxana Matran

**Affiliations:** 1 Department of Pediatrics, “Carol Davila” University of Medicine and Pharmacy 020021, Bucharest, Romania; 2 Department of Pediatrics, “Grigore Alexandrescu” Emergency Hospital for Children, Bucharest, Romania; 3 Department of Physiology, “Carol Davila” University of Medicine and Pharmacy 020021, Bucharest, Romania

**Keywords:** chest tube drainage, parapneumonic effusion, pleural empyema, thoracic ultrasound, video-assisted thoracoscopy

## Abstract

**Background/objectives:**

In children and infants, thoracic empyema most often develops as a complication of parapneumonic pleural effusions progressing to purulent collections. With an estimated incidence of approximately 0.6% among pediatric pneumonia cases, empyema remains associated with significant morbidity. This study aimed to characterize pediatric parapneumonic empyema by analyzing clinical, biochemical, and radiological parameters and their relationship with treatment decisions and outcomes.

**Materials and methods:**

We conducted a retrospective, single-center study including children diagnosed with parapneumonic pleural empyema and treated in the Pulmonology Department of the “Grigore Alexandrescu” Emergency Hospital for Children between January 2021 and December 2024. Only patients managed surgically—either by chest-tube drainage or video-assisted thoracic surgery (VATS)—were included. Fibrinolytic therapy was not used due to limited institutional experience, with VATS preferred in complicated cases. Patients were stratified according to initial intervention. Clinical, laboratory, and imaging data were extracted from medical records.

**Results:**

A total of 33 patients were included, with a median age of 4 [3–8] years. The median time to initial intervention was 2 [1–5] days. Fourteen patients (42.4%) underwent primary VATS after a median of 4.5 [2–6.3] days, while 19 received initial chest-tube drainage after 1 [0–3] days, with a mean drainage duration of 21.2 ± 11.5 days. Median hospital stay for the cohort was 27 [21–38.5] days. Loculations and septations were significant predictors of hospitalization length. Drainage duration was significantly shorter in the primary VATS group compared with the chest-tube group (9.5 [7.8–12.5] vs. 19 [11–30] days; *p* = 0.011). Dyspnea strongly predicted selection of VATS as initial treatment (OR 18.0, 95% CI 1.86–174.21; *p* = 0.013). Imaging findings on thoracic ultrasound did not significantly influence the choice of initial intervention. Computed tomography, performed in 45.5% of cases, identified complications such as bronchopleural fistula, empyema necessitans, and pyopneumothorax, and was associated with prolonged hospitalization.

**Conclusions:**

Chest-tube drainage was the most frequent initial treatment, with escalation decisions driven primarily by clinical presentation rather than imaging or biochemical markers. Thoracic ultrasound was valuable for assessing effusion complexity but had limited prognostic utility. The lack of fibrinolytic therapy resulted in a high rate of VATS, highlighting the need for standardized, symptom-driven management algorithms integrating clinical, laboratory, and imaging data.

## 1 Introduction

Under normal conditions, pleural fluid is maintained at approximately 0.3 mL/kg by balanced production and lymphatic drainage; infection disrupts this balance by increasing vascular permeability and triggers cytokine mediated coagulation resulting in excessive fibrin production, septations, loculations, and pleural adhesions, that impede fluid resolution (1). When pleural effusions is becoming purulent, they are converting into empyema, most commonly as a complication of parapneumonic effusions (2). Empyema occurs in 0.6% pneumonia cases with an estimated incidence of 3.3 cases per 100 000 children (3). Despite a decline in the incidence of community-acquired pneumonia following the introduction of pneumococcal vaccines, pleural empyema remains a significant complication due to factors such as bacterial resistance and virulence (4). The most common causative agents include *Streptococcus pneumoniae, Streptococcus pyogenes*, *Staphylococcus aureus* and *Haemophillus influenzae* (5). Hamm and Light described three progressive stages of parapneumonic effusion—exudative, fibrinopurulent, and organizational—each marked by increasing fluid complexity and specific intervention needs (6).

Chest radiography is the first-line tool for detecting pleural effusion but cannot confirm empyema or differentiate effusion types, often requiring further imaging (e.g., ultrasound or CT) for diagnosis, especially since loculated effusions may mimic lung abnormalities (7). CT is not routinely recommended for pediatric pleural effusions, but when used, IV contrast is essential for optimal pleural imaging. It detects pleural thickening, extrapleural fat density, and loculations, but may miss thin septations unless air is present (e.g., post-thoracentesis or pneumothorax). Despite this, CT aids in differentiating empyema from abscess, diagnosing pyopneumothorax/fistulae, and guiding procedures like VATS (8, 9). Thoracic ultrasound (TUS) serves as the primary imaging tool for pleural effusions, allowing detection, volume estimation and visualization of features like fibrinous strands, fibrous septations, loculations (non-free-flowing fluid pockets), pleural thickening, and—in advanced stages—impaired lung mobility, while offering real-time radiation-free assessment, essential for disease staging and guiding interventions (1). Very recent studies emphasize the role of TUS in neonatal practice in identifying the need for respiratory support or surfactant administration (10) while significantly reducing the radiation exposure in ventilated patients (11). One of the most important parameters assessed by thoracic ultrasound is the presence of septations, which when numerously, are possible indicators of clinical outcomes (12). TUS is an essential tool for guiding pleural drainage decisions in parapneumonic effusions. Loculated or echogenic fluid observed on ultrasound strongly supports the need for drainage, as these findings typically indicate complex effusions that are unlikely to resolve spontaneously. Conversely, free anechoic fluid requires further evaluation to avoid inappropriate therapeutic interventions, as it may represent uncomplicated effusion or transudate rather than empyema (13).

Contemporary management of empyema includes both conservatory options represented by antibiotics alone or in combination with thoracocentesis and interventions like chest tube drainage with or without fibrinolytic agents and surgical interventions like video-assisted thoracoscopy (VATS) or open-drainage techniques such as thoracotomy and decortication (14, 15). In a recent meta-analysis of treatment options for empyema in children, the authors reported that when compared with chest-tube drainage alone, chest-tube drainage with fibrinolytics, VATS and thoracotomy were associated with shorter hospitalization time, with no significant differences between the last three treatment modalities (16).

To our knowledge, this is the first Romanian study designed to comprehensively analyze the demographic, clinical and paraclinical profiles of pediatric patients with empyema. The study aims to evaluate the usefulness of available diagnostic investigations, including TUS, laboratory markers and advanced imaging techniques, in guiding therapeutic decision-making and establishing outcome-related prognostic factors. A secondary objective was to assess whether there are differences in initial therapeutic interventions and in the clinical course of patients with empyema.

## 2 Materials and methods

This was a retrospective single center study comprising patients diagnosed with parapneumonic pleural empyema, treated in the Pulmonology Department of “Grigore Alexandrescu” Emergency Hospital for Children, Bucharest, Romania between January 2021-December 2024. We selected patients who were treated surgically, either with a simple pleural drain or VATS. None of the patients received fibrinolytics, due to limited experience with their use in our department, VATS being the technique of choice in complicated cases of empyema. Patients managed conservatively (systemic antibiotics only) were excluded. All clinical, laboratory, and radiological data were extracted retrospectively from the institutional electronic medical records. Data extraction was performed independently between January and February 2025 by E.R.M and A.U with discrepancies resolved by senior investigator A.I.U. Demographic parameters were recorded for all participants.

The study was approved by the Ethics Committee of our Institution (reference number #10003/1.04.2025). Our research was designed and reported in accordance with the Strengthening the Reporting of Observational Studies in Epidemiology (STROBE) guidelines, and the completed STROBE checklist is provided as a supplemental file.

Given the relatively rare incidence of pediatric empyema and the retrospective nature of our study, our sample size was determined by the total number of eligible cases treated during the study period, rather than by *a priori* power analysis. While a formal power calculation was not performed prior to data collection, it is, for sure, important to note that studies with small sample sizes, such as ours, may be underpowered to detect small or moderate effect sizes, and any statistically significant findings are more likely to reflect large effect sizes. Our single-center cohort reflects the real-world constraints of a rare disease population, and the sample size represents all available cases within the specified timeframe.

Patients were divided by initial treatment into chest-tube drainage-group or VATS-group. Early VATS was defined as intervention performed within 5 days of hospitalization whether as primary or secondary treatment.

Collected clinical and laboratory variables included: radiological parameters such as TUS assessment of fluid - maximum thickness (mm), presence of septation (defined by the presence of echogenic linear structures within the pleural fluid, indicative of empyema organization) or loculation (non-mobile liquid pockets with septations ≥2 mm, suggesting an advanced evolutive state), anatomic localization (right-/left-sided, bilateral). Clinical symptoms such the presence of dyspnea (defined as presence of at least two of the following: use of accessory muscles, nasal flaring, grunting and chest retractions), cough and thoracic pain were noted. Documented biochemical parameters were peripheral blood leukocyte count (cells/mm^3^), platelet count (cells/mmc), serum C-reactive protein concentration (CRP, mg/dL) and serum procalcitonin (ng/mL).

CT scans were indicated in patients with large or atypical pleural effusions, suspicion of complications (e.g., loculations, empyema), or insufficient response to initial therapy.

Additional data included time until intervention (days), prior antibiotics administration, the duration (days) of chest-tube drainage and time (days) to VATS conversion for those treated initially with chest-tube drainage. Also, need for reintervention after an initial VATS and association of complications such as pyopneumothorax, *empyema necesitans* and broncho-pleural fistula were documented, as described on the CT-scan.

Length of hospitalization (days) and in-hospital antibiotic treatment strategies (association of 2,3 or 4 concurrent antibiotics ≥72 h) were documented for all patients.

All thoracic ultrasound examinations were conducted by board-certified radiologists, each possessing a minimum of five years of dedicated experience in thoracic imaging. These examinations were performed either upon patient admission or at the point when clinical indicators suggested escalating severity in cases of pneumonia accompanied by minimal pleural fluid. The procedures were carried out within the radiology department, as our institution ensures continuous, 24/7 availability of an experienced radiologist, thereby obviating the need for point-of-care-ultrasound (POCUS) performed by non-radiologist clinicians. In our country, the utilization of POCUS in pediatric emergency hospitals is less common compared to other countries, primarily due to the consistent availability of radiology rounds.

Microbiological etiology was not incorporated into the primary analysis due to the high rate of negative cultures, most likely secondary to prior empirical antibiotic therapy before pleural sampling, which limited pathogen identification and precluded robust statistical comparisons.

### 2.1 Statistical analysis

Data were analyzed using SPSS v26 (Chicago, IL, USA). Descriptive statistics were calculated for continuous and categorical variables. The normality of continuous variables was assessed using the Shapiro–Wilk test. Depending on the distribution of the data, continuous variables were summarized as mean ± standard deviation (SD) or median with interquartile range (IQR). Categorical variables were expressed as frequencies and percentages. Associations between categorical variables were analyzed using the chi-square test or Fisher's exact test, depending on cell frequencies. Comparisons of continuous variables between groups were performed using the Mann–Whitney U test or Independent Samples t-test, based on data distribution. Linear and logistic regression models were used to assess predictors of specific outcomes.

Continuous variables (e.g., age, laboratory parameters, time to intervention, and length of hospitalization) were assessed for normality and expressed as mean ± standard deviation or median (IQR), as appropriate; between-group comparisons were performed using the Student's t-test or Mann–Whitney U test accordingly, while categorical variables (e.g., sex, clinical features, radiological findings, and treatment characteristics) were analyzed using the chi-square test or Fisher's exact test, as appropriate.

A *p*-value of <0.05 was considered statistically significant.

## 3 Results

Initial study population consisted of 36 children; 1 child was excluded due to insufficient data and 2 were excluded because of conservatory (only systemic antibiotics) treatment. The final study cohort included 33 patients, 16 (48.5%) boys and a median [IQR] age at inclusion of 4 [3–8] years. Fourteen (42.4%) of children undergo VATS as initial intervention, *p* = 0.384. Median time until initial intervention for the entire cohort was 2 [1–5] days. Characteristics of the study population are described in Table 1.

**Table 1:** Characteristics of study population.

	Type of initial intervention		
	VATS (*n* = 14)	Chest-tube drainage (*n* = 19)	*p*-value
Sex [*n* (%)]			0.393
Male	8 (57.1)	8 (42.1)	
Female	6 (42.9)	11 (57.9)	
Residence [*n* (%)]			0.024
Urban	10 (71.4)	6 (31.6)	
Rural	4 (28.6)	13 (68.4)	
Median age at inclusion, years [IQR]	4.5 [3.8–8]	4 [2–8]	0.439
Median time until intervention [IQR] (days)	4.5 [2–6.3]	1 [0–3]	0.004
Mean number of leukocytes ± SD (cells/mm^3^)	24,317.9 ± 9,825	23,025.3 ± 8,734.1	0.699
Mean number of platelets ± SD (cells/mm^3^)	346 × 10^3^ [230 × 10^3^–476 × 10^3^]	362 × 10^3^ [248 × 10^3^– 489 × 10^3^]	0.760
Mean serum C-reactive protein level ± SD, (mg/dL)	26.3 ± 13	18.7 ± 9.7	0.079
Median serum procalcitonin level^a^, ng/mL [IQR]	4.8 [0.44–8.5]	1.1 [0.5–4]	0.134
Median thickness of pleural fluid [IQR] (mm)	33 [23.3–50]	31 [18–40]	0.843
Septations			0.095
Yes	10 (71.4)	8 (42.1)	
No	4 (28.6)	11 (57.9)	
Loculations			0.284
Yes	7 (50)	6 (31.6)	
No	7 (50)	13 (68.4)	
Necrosis			0.510
Yes	3 (21.4)	3 (15.8)	
No	11 (78.6)	16 (84.2)	
Median length of hospitalization [IQR], (days)	27 [20–35.5]	24 [22–42]	0.815
Cough			0.676
Yes	13 (92.9)	18 (94.7)	
No	1 (7.1)	1 (5.3)	
Thoracic pain			0.461
Yes	4 (28.6)	15 (78.9)	
No	10 (71.4)	4 (21.1)	
Number of antibiotics [*n* (%)]			0.883
Two	1 (7.1)	2 (10.5)	
Three	3 (21.4)	3 (15.8)	
Four	10 (71.5)	14 (73.7)	
Dyspnoea [*n* (%)]			0.005
Yes	7 (50)	18 (94.7)	
No	7 (50)	1 (7.7)	
Anatomic distribution [*n* (%)]			0.454
Right-sided	6 (42.9)	7 (36.8)	
Left-sided	8 (57.1)	10 (52.6)	
Bilateral	0	2 (10.5)	
Previous antibiotic therapy [*n* (%)]			0.652
Yes	3 (21.4)	4 (21.1)	
No	11 (78.6)	15 (78.9)	

a Computed for 30 patients; VATS, video-assisted thoracoscopy.

**Table 2:** Biochemical profile stratified by thoracic ultrasound parameters.

	Parameter			
TUS categories	Leucocytes (cells/mm^3^), mean ±SD	Platelets (cells/mm^3^) median [IQR]	CRP (mg/dL) mean ±SD	Procalcitonin^a^ (mg/mL) median [IQR]
Loculations				
Yes (*n* = 20)	24,421.5 ± 8,782.5	351 × 10^3^[218 × 10^3^−798 × 10^3^]	23.4 ± 13.3	2.5 [0.4–7]
No (*n* = 20)	23,022.5 ± 9,460.4	363.5 × 10^3^[278.5 × 10^3^−454.8 × 10^3^]	21 ± 10.7	0.2 [0.5–4.8]
*p* value	0.668	1	0.585	0.350
Septations				
Yes (*n* = 18)	25,601.7 ± 10,067.3	374.5 × 10^3^[245.8 × 10^3^−557.25 × 10^3^]	21.9 ± 11.5	1.1 [0.4–7.9]
No (*n* = 15)	21,140 ± 7,346.8	299 × 10^3^ [248 × 10^3^−420 × 10^3^]	22 ± 12.3	3.4 [0.6–4.8]
*p* value	0.164	0.585	0.977	0.509
Pleural fluid thickness				
>25 mm (*n* = 22)	24,451.4 ± 10,113	356 × 10^3^ [226.8 × 10^3^−468 × 10^3^]	24.2 ± 9.8	2 [0.4–6.7]
<25 mm (*n* = 11)	21,818.2 ± 6,655.2	362 × 10^3^ [299 × 10^3^−597 × 10^3^]	17.5 ± 14.2	2 [0.5–4.7]
*p* value	0.379	0.462	0.172	0.756

CRP, C-reactive protein; IQR, interquartile range; SD, standard deviation; TUS, thoracic ultrasound.

a Computed for 30 patients.

Statistical analysis identified platelet count (B=+8,073 × 10⁻⁶, *p* = 0.003) and CRP levels (B = 0.179, *p* = 0.009) as significant predictors of time to primary intervention, though the model explained only 37.7% of variance (adjusted R^2^ = 0.233, F = 2.619, *p* = 0.04). Leukocyte count (*p* = 0.400) and TUS parameters—fluid thickness (*p* = 0.501), septations (*p* = 0.109), and loculations (*p* = 0.105)—showed no significant links. Introducing procalcitonin rendered the model statistically non-significant.

In another 8 (42.1%) patients VATS was performed after initial chest-tube drainage treatment, after a mean time of 11.5 ± 6.6 days. TUS parameters were not associated with treatment failure, *p* = 0.506 for loculations, *p* = 0.551 for septations and *p* = 0.492 for pleural fluid thickness.

**Figure 1:**
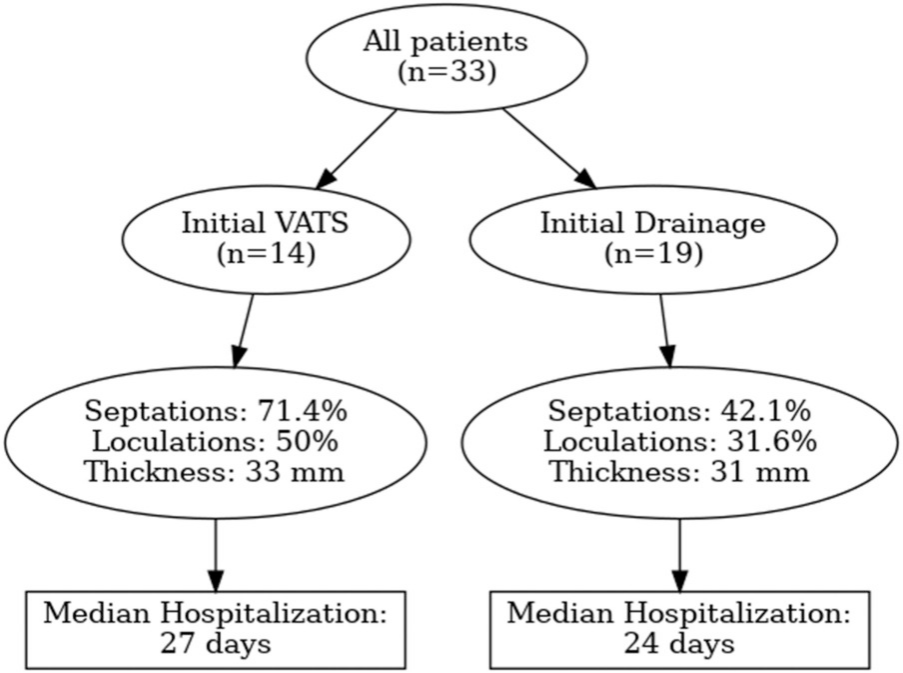
Decision -making flow-chart regarding primary intervention type and outcome. VATS, video-assisted thoracoscopy.

We evaluated TUS parameters for their ability to predict the primary intervention type (VATS vs. chest tube drainage). None of the TUS parameters—pleural fluid thickness (OR 1.00, 95% CI 0.966–1.051; *p* = 0.734), septations (OR 3.49, 95% CI 0.548–22.189; *p* = 0.186), or loculations (OR 1.06, 95% CI 0.176–6.433; *p* = 0.947)—demonstrated statistically significant associations with intervention choice (Figure 1). CRP exhibited a marginal inverse association with surgical intervention (OR 0.92, 95% CI 0.85–1.00; *p* = 0.041). The presence of dyspnea emerged as a robust predictor, accounting for 33% of the variance in intervention selection (Nagelkerke R^2^ = 0.33). Dyspnea increased the likelihood of VATS by 18-fold compared to chest tube drainage (OR 18.00, 95% CI 1.86–174.21; *p* = 0.013).

Patients were equally divided between early and late VATS intervention groups, each comprising 11 (50%) children. Septation identified on TUS were significantly associated with early intervention (*p* = 0.04) whereas loculations were not predictive of VATS within the first 5 days of hospitalization (*p* = 0.087).

In the VATS cohort (*n* = 22), reintervention was necessary in 22.7% (*n* = 5) of children. Septations were not significantly associated with reintervention risk (OR 2.75, 95% CI 0.36–21.1; *p* = 0.333). Similarly, loculations were not predictive of reintervention (OR 0.47, 95% CI 0.06–3.5; *p* = 0.46), nor was pleural fluid thickness (OR 1.005, 95% CI 0.94–1.07; *p* = 0.89). Furthermore, VATS timing did not significantly influence reintervention risk (OR 0.59, 95% CI 0.08–4.5; *p* = 0.613).

Median duration of drainage was 12 [9–23.5] days for entire study group. The duration of drainage differed significantly between the two groups, with the initial VATS group (*n* = 14) having a median drainage duration of 9.5 [7.8–12.5] days compared to 19 [11–30] days in the initial chest tube drainage group (*n* = 19), *p* = 0.011. A linear regression model (R^2^ = 0.357, adjusted R^2^ = 0.143, F = 2.918, *p* = 0.051) identified loculations (B = + 16.02, *p* = 0.014) and septations (B = −14.9, *p* = 0.014) as significant predictors of drainage duration while pleural fluid thickness (B = + 0.148, *p* = 0.228) showed no significant association with drainage duration into entire cohort. A separate regression model evaluating biochemical parameters, including leukocyte count, platelet count, CRP, and procalcitonin, showed no predictive value for drainage duration (R^2^ = 0.287, adjusted R^2^ = 0.082, F = 0.562, *p* = 0.692), with individual *p*-values as follows: leukocytes (*p* = 0.504), platelets (*p* = 0.496), CRP (*p* = 0.856), and procalcitonin (*p* = 0.259).

Median hospitalization length was 27 [21–38.5] days for the overall cohort. A linear regression model (R^2^ = 0.159, adjusted R^2^ = 0.159, F = 3.02, *p* = 0.046) identified loculations (B = + 14.27, *p* = 0.010) and septations (B = −12.58, *p* = 0.020) as significant predictors of hospitalization length. Pleural fluid thickness showed no significant association (*p* = 0.435). However, adding laboratory values (*p* = 0.257) and initial intervention type (*p* = 0.360) reduced the model's validity, leading to a loss of statistical significance. Supplementary, performance of VATS at any point did not significantly change the hospitalization length, 28 [20.8–44.5] days vs. 28 [22–35] days for the chest-tube drainage only group, *p* = 0.665. Pleural fluid thickness did not correlate with hospitalization length (Spearmen's *ρ* = 0.278, *p* = 0.118), mean number of leukocytes (*ρ* = 0.066, *p* = 0.715), platelets (*ρ* = −0.040, *p* = 826), C-reactive protein (*ρ* = 0.267, *p* = 0.154) nor the procalcitonin levels (*ρ* = 0.235, *p* = 0.189). Biochemical markers, including leukocyte count, platelet count, CRP, and procalcitonin levels, showed no significant correlation with hospitalization length (Spearman's *ρ* = −0.170, *p* = 0.344 for platelets; *ρ* = −0.119, *p* = 0.511 for leukocytes; *ρ* = 0.178, *p* = 0.321 for CRP; *ρ* = 0.062, *p* = 0.744 for procalcitonin.

Early VATS intervention did not significantly reduce median hospitalization length, with the early VATS group demonstrating 29 days [IQR 20–29] vs. 31 days [IQR 27–49] in the late VATS group (*p* = 0.151). A linear regression model incorporating VATS timing explained minimal variance in hospitalization duration (R^2^ = 0.042), with no statistically significant association observed between VATS timing and length of stay (coefficient B = −5.6, *p* = 0.358). Additionally, VATS performed at any time did not reduce hospitalization length compared to chest-tube drainage alone (24 [22–35] days vs. 28 [20.8–44.5] days, *p* = 0.665.

**Figure 2:**
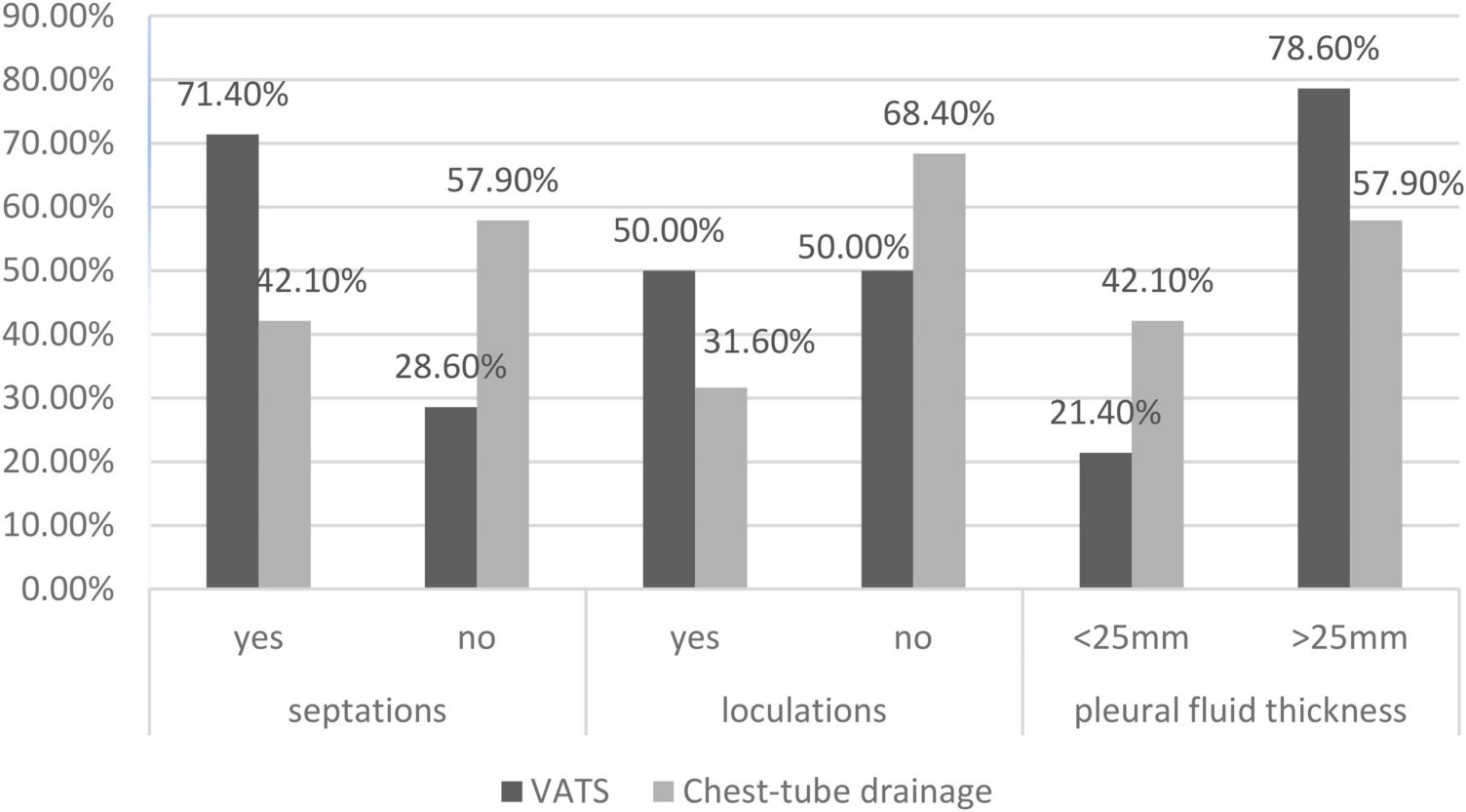
Thoracic ultrasound features and distribution based on type of primary intervention. VATS, video-assisted thoracoscopy.

Biochemical parameters, including leukocyte and platelet counts, as well as inflammatory markers such CRP and procalcitonin, were comparable across the categories defined by TUS parameters (Figure 2).

Distribution of TUS findings according to type of primary intervention is represented in Figure 2.

CT was performed in 15 children (45.5%). Complications such as bronchopleural fistula, empyema necessitans, and pyopneumothorax were observed in 7 (21.2%), 4 (12.1%), and 7 (21.2%) children, respectively, with 5 children (15.2%) experiencing more than one complication. CT imaging was associated with a significantly prolonged median hospitalization duration (35 days [IQR 25–49] in the CT group vs. 24.6 days [IQR 20–27.8] in the non-CT group; *p* = 0.001). Complications occurrence was irrespective of type of initial treatment (28.6% for VATS group and 47.4% in the simple drainage group, *p* = 0.233). Additionally, within the VATS-at-any-point group, intervention timing showed no significant association with the occurrence of any complication (*p* = 0.38).

## 4 Discussion

To our knowledge, this is the first study that comprehensively assess the utility of TUS in predicting the therapeutic decision and the potentially evolutive outcomes of pleural empyema in children, in Romania. Although it makes an important contribution to the specialized literature, our study has several limitations. First, the retrospective data collection may have introduced variability due to the non-uniform nature of the data sources from medical records. Second, the absence of specific guidelines to standardize clinical practice, has led each physician to adopt a personal-approach to therapeutical and diagnostics decision-making, influenced by individual experience and the availability of certain interventions at the time of patient admission. Moreover, personal-approach to therapeutic management, particularly antibiotic regimens, may have impacted patient outcomes. In addition, we recognize that other potential confounding factors may have impacted patient outcomes in our cohort. These include the administration of adjunctive medications (such as antipyretics or non-steroidal anti-inflammatory drugs). Moreover, institutional practices and adherence to clinical guidelines may vary, further contributing to heterogeneity in patient management and outcomes. As our study was retrospective and observational in nature, it was not possible to fully control for these confounders, which should be considered when interpreting the results. Finally, the lack of standardized ultrasound evaluation criteria and operator dependency could have led to subjective and non-uniform interpretations of TUS findings. As a statistical limitation of this study is the relatively small sample size and single-center retrospective design, which may majorly restrict the generalizability of the findings.

The main findings of this study indicate therapeutic decisions in pediatric pleural empyema are primarily guided by clinical parameters suggestive of respiratory distress, rather than imaging or laboratory results. In particular, dyspnea was a significant predictor of VATS, whereas TUS findings and inflammatory biomarkers such as CRP and procalcitonin showed limited influence.

Our findings align with Long et al.'s “less may be best” approach, with chest tube drainage as the initial intervention in more than half of cases (17). Despite the historical importance of loculated pleural fluid as an indicator for surgical management, as described by Shankar et al. (18), our logistic regression analysis found no significant associations between TUS parameters—pleural fluid thickness, septations, or loculations—and the initial intervention type. While septations were not associated with the choice between chest tube drainage and VATS, they were linked with early VATS use, suggesting a role as an indicator of disease progression rather than a primary determinant of intervention.

While septations alone are not associated with clinically significant outcomes, they do play a role in influencing initial treatment decisions, often favoring surgical intervention (19), contrary to our findings. Similarly, Stevic et al. report that in predominantly septated effusions, open thoracotomy and decortications are more frequently used (20). In our study, septations were not associated with type of primary intervention but with early VATS suggesting that this pattern is an indicator of disease progression rather than a direct determinant of intervention choice. The discrepancy underscores that, in our setting, intervention decisions are multifactorial, shaped not only by imaging and clinical features but also by institutional practices and surgeon experience. This highlights the need for standardized decision-making tools that integrate clinical, radiological, and laboratory indicators.

Reintervention rates in our study were higher than previously reported by Freitas et al., who found reintervention in 12% of patients. In their study, VATS was the initial procedure, imposed by the presence of septations in the pleural fluid (21).

Regarding laboratory findings, CRP showed a marginal inverse association with surgical intervention, while platelet count predicted shorter time-to-intervention. The predictive capacity of platelet count for time to intervention may be attributed to platelets' role in promoting pleural fibrin deposition through the release of thrombospondin-1 (a platelet-derived glycoprotein that stabilizes fibrin matrices) and plasminogen activator inhibitor-1 (PAI-1), which inhibits fibrinolysis, thereby accelerating pleural organization (19). However, the predictive capacity of inflammatory markers like CRP and procalcitonin was limited, possibly due to multicollinearity or their reflection of systemic rather than localized disease. Notably, CRP and dyspnea were the only parameters associated with the type of intervention, with elevated CRP correlating with shorter time to VATS.

The lack of significant correlations between laboratory values (CRP, leukocyte/platelet counts) and hospitalization length aligns with evidence showing these markers poorly predict clinical outcomes in empyema management. Our findings align with those of Medeiros et al. supporting the fact that perioperative CRP levels did not correlate with clinical outcomes (22). Additionally, the lack of correlation between loculations, septations and pleural fluid thickness observed on TUS and paraclinical parameters, including leukocytosis, thrombocytosis, and elevated CRP levels, suggests that the severity of systemic inflammation does not necessarily align with the ultrasonographic characteristics of pleural fluid, suggesting a potential lack of concordance between systemic inflammatory responses and localized pleural pathologic processes.

While Shirota et al. demonstrated comparable efficacy between drainage + fibrinolytics and VATS (23), our protocol omitted fibrinolytics, resulting in 42.1% requiring escalation to VATS—a disparity likely attributable to this therapeutic difference but also, as stated by Goldin et al., it is common for a child to undergo both interventions, based on disease progression (24). In our institution, fibrinolytic agents were not used in the management of pediatric empyema during the study period. This decision was based on the limited experience of our clinical team with fibrinolytic therapy in this specific context, as well as the absence of established local protocols for their safe administration. As a result, all patients included in this cohort were managed either with chest tube drainage or VATS. We acknowledge that the omission of fibrinolytics may have influenced the treatment escalation rate and overall outcomes, and this represents an important limitation of our study. Future efforts to establish standardized protocols and training in the use of fibrinolytics may help broaden the therapeutic options available in our center and align our practice with current international guidelines.

Consistent with Haggie et al., TUS parameters like septations and loculations lacked predictive value for treatment failure. Hyperchogenicity was excluded from our TUS analysis due to inconsistent data (25).

The British Thoracic Society (BTS) guidelines for pediatric pleural infection management in children, emphasize that while TUS cannot reliably determine the stage of pleural infection, it is effective for estimating effusion size, distinguishing free from loculated fluid and assessing fluid echogenity (26). CT is reserved for exceptional circumstances and not recommended as a routine diagnostic tool (1). Furthermore, CT becomes essential when surgical intervention, such as VATS is indicated (27). In our study, nearly half of the patients required CT-scan examination, primarily due to the severity of the disease and concerns about potential complications, supporting literature data.

In terms of clinical outcomes, empyema is associated with increased length of hospitalization (28). Our cohort experienced longer hospitalization duration compared to findings from other studies. Jeniebi et al. report shorter hospital stays following the implementation of pediatric pleural empyema guidelines from 15 [5–32] days to 11 [6–27] days (29). Comparable (although shorter) to our study, Marhueda et al. reported similar median hospital stay of 14 respectively 13 days for the VATS group and chest-tube drainage with urokinase group; moreover, median post-operative stay and mean febrile duration were similar between the two groups (30). Longer hospital length in our hospital may be explained first by study population profile, etiology of pleural infection, the choice of antibiotics regimens, conservative management practices in our Pulmonology Department and delayed escalations to VATS. Furthermore, in our study, VATS performed at any stage of management did not reduce hospitalization length, conflicting with the conclusions of Redden et al. whose meta-analysis supported surgical intervention for shortening hospital stays in pleural empyema (31). On the contrary, Schultz et al., report that VATS treatment reduced the hospitalization length and the number of days with fever (32). In a systematic review of literature, Avansino et al. (33), indicate a lower hospitalization length associated with primary VATS intervention. However, the same authors (33) report shorter tube thoracostomy time for primary operative group (4.4 vs. 10.6 days), similar to our study. More recent data is also indicative of shorter hospitalization time for VATS treated patients (16, 34, 35). In our population study, hospitalization length was not associated with TUS parameters as supported by other authors (27).

Contrary to the findings of Di Mitri et al. early VATS in our cohort did not demonstrate improved outcomes, particularly regarding hospitalization length and occurrence of complications (4).

Total duration of drainage was shorter for the primary VATS group, comparative with the observations of Ratta et al., who report reduced drainage duration in VATS group (36), although shorter than the ones in our study. Loculations were associated with prolonged drainage duration, likely indicative of complex effusions necessitating extended therapeutic management. Conversely, septations correlated with shorter drainage duration, potentially reflecting earlier intervention practices as observed in our cohort. Inflammatory markers, such as CRP and procalcitonin, likely reflect systemic disease severity rather than localized pleural fluid organization. Variables such as antibiotic selection, prior antibiotic use, and biomarker trends over time (e.g., rising/falling CRP) may hold greater prognostic value than isolated admission values. This last finding is supported by the observations of Tsurono et al. which indicated that the utility of CRP measured in the 3rd day of hospitalization may predict the need for surgical intervention in adult patients with empyema (37). Moreover, Carboni et al. (38) reported that CRP and procalcitonin exhibit specific pre- and postoperatively patterns, with peak values in day 2. Undoubtedly, additional clinical and therapeutic factors influence outcomes, necessitating validation in larger, prospective cohorts. In our study, baseline CRP measurement was made on the first day of admission.

Consistent with the findings of Marhuenda et al., our study demonstrated that the duration of chest tube placement was significantly shorter in patients who underwent initial VATS compared to those managed with chest tube drainage alone, despite the overall drainage duration being lower across both groups (30). The shorter drainage duration in the VATS group likely reflects the mechanical advantages of VATS over passive drainage and suggests that standardized protocols for early VATS in select cases (e.g., septated effusions) may optimize outcomes.

Given that septations are associated with early-VATS, platelet count and CRP correlate with reduced time-to-intervention, and CRP and dyspnea are associated with type of intervention, namely VATS, future perspectives may be represented by development of a scoring system by integrating clinical, radiological and laboratory parameters to guide interventions decision-making.

## 5 Conclusions

While TUS remains indispensable for empyema diagnosis, its prognostic utility—like that of biochemical markers —proved limited in predicting therapeutical and clinical trajectories. As reflected in current clinical guidelines, the most appropriate treatment approach for pediatric empyema is guided by a combination of clinical signs, imaging features, laboratory markers, and evolution under initial therapy. Our study underlines the importance of individualized treatment strategies, tailored to each patient's presentation and response to initial management.

## Data Availability

The raw data supporting the conclusions of this article will be made available by the authors, without undue reservation.
